# Trends and factors associated with initiation of HIV treatment among PLHIV in Jamaica, 2015–2019

**DOI:** 10.1371/journal.pone.0265468

**Published:** 2023-05-26

**Authors:** Anya Cushnie, Ralf Reintjes, J. Peter Figueroa, Miia Artama

**Affiliations:** 1 Faculty of Social Sciences, Unit of Health Sciences, Tampere University, Tampere, Finland; 2 Department of Health Sciences, Hamburg University of Applied Sciences, Hamburg, Germany; 3 Department of Community Health and Psychiatry, University of the West Indies, Mona, Jamaica; Lagos State University, NIGERIA

## Abstract

**Introduction:**

Jamaica did not achieve the UNAIDS 90-90-90 targets in 2020. This study aimed to examine trends and factors associated with uptake of HIV treatment among people living with HIV (PLHIV) in Jamaica and to assess the effectiveness of revised treatment guidelines.

**Methods:**

This secondary analysis used patient-level data from the National Treatment Service Information System. The baseline sample was 8147 PLHIV initiating anti-retroviral treatment (ART) between January 2015-December 2019. Descriptive statistics were used to summarize demographic and clinical variables and the primary outcome timing of ART initiation. Multivariable logistic regression was used to assess factors associated with ART initiation (same day vs 31+ days), using categorical variables for age group, sex and regional health authority. Adjusted odds ratios and 95% confidence intervals are reported.

**Results:**

Most persons initiated ART at 31+ days (n = 3666, 45%) after the first clinic date or on the same day (n = 3461, 43%). Same day ART initiation increased from 37% to 51% over 5 years and was significantly associated with males (aOR = 0.82, CI = 0.74–0.92), 2018 (aOR = 0.66, CI = 0.56–0.77), 2019 (aOR = 0.77, CI = 0.65–0.92). late HIV diagnosis (aOR = 0.3, CI = 0.27–0.33) and viral suppression at the first viral load test (aOR = 0.6, CI = 0.53–0.67). ART initiation at 31+days was associated with 2015 (aOR = 1.21, CI = 1.01–1.45) and 2016 (aOR = 1.30, CI = 1.10–1.53) compared to 2017.

**Conclusion:**

Our study shows that same day ART initiation increased between 2015–2019, however it remains too low. Same day initiation was associated with the years after Treat All implementation and late initiation before Treat All, providing evidence of the strategy’s success. In order to achieve the UNAIDS targets, there is a need to also increase the number of diagnosed PLHIV retained on treatment in Jamaica. Further studies should be conducted to understand important challenges to accessing treatment as well as differentiated care models to improve treatment uptake and retention.

## Introduction

In 2019, The Caribbean region continued to have the second highest regional rate of HIV prevalence and second highest ratio of female to male cases after Sub-Saharan Africa [1]. With regards to the UNAIDS global 90-90-90 targets which aimed to diagnose 90% of persons living with HIV (PLHIV), retain 90% of PLHIV diagnosed on antiretroviral therapy (ART) and ensure that 90% of people receiving ART are virologically suppressed by 2020 [2], the Caribbean region did not achieve these targets with 77% diagnosed, 81% initiated and retained on treatment and 80% virologically suppressed [3]. Compared to the global achievement of 81%-85%-88% [4], the Caribbean is making progress slowly; some countries have achieved elements of the global targets [3] but others are lagging. Similarly in 2019, Jamaica had an adult HIV prevalence of 1.5% and an estimated 32,000 persons living with HIV (PLHIV) [5] while achievement of the UNAIDS targets stood at 85%-52%-66% [6]. Jamaica is lagging considerably in achieving treatment retention and viral suppression targets and did not achieve the UNAIDS targets in 2020 [7].

The Government of Jamaica has provided public access to HIV treatment since 2004 [5]. There are forty-four HIV treatment sites across the island which deliver treatment and care services to both adults and children living with HIV [8]. Jamaica adopted the World Health Organization’s (WHO) Treat All strategy in January 2017 [9] and a key objective is same day initiation of ART following HIV diagnosis, regardless of clinical status [10,11]. Despite these efforts to broaden access, many PLHIV are still not on treatment [12]. Some of the factors that have contributed to low ART uptake and retention include discriminatory practices in public facilities and at home [13], having no food to take medication or running out of medication [14].

To improve treatment outcomes and achieve the 95-95-95 targets, the Ministry of Health and Wellness (MoHW) implemented the World Health Organization’s recommended Treat All (or Test and Start) strategy in 2017 [5]. Treat All aims for immediate ART initiation regardless of HIV clinical stage to achieve viral suppression sooner [15]. Randomized trials have demonstrated multiple benefits of rapid ART initiation compared to later ART start, including improved ART uptake, increased retention in care, higher rates of viral suppression and reduced risk of mortality [16,17]. Trials conducted in Haiti and South Africa demonstrated increased retention rates between 79%-81% for patients in same-day ART initiation groups compared to standard ART initiation groups [18,19].

The National HIV Strategic Plan recommends an analysis of factors associated with ART uptake in order to improve treatment outcomes. This study aims to describe PLHIV who accessed ART and assess factors associated with treatment uptake. This is the second paper in a series of manuscripts that aim to assess HIV treatment outcomes for Jamaica based on demographic and clinical factors as well as assess the implementation of Treat All. In a previous paper we demonstrated associations between routinely collected demographic variables and HIV stage of diagnosis and viral load status [20].

## Methods

This secondary analysis uses the National Treatment Site Information System (TSIS2). TSIS2 is a centralized case-based database, governed by the MoHW and currently used by all public, non-governmental and some private HIV treatment facilities in Jamaica. This dataset did not include private providers. TSIS2 stores patient level demographic and clinical data using over 100 variables.

The MoHW Internal Review Board provided ethical approval (Study No: 2017/20). The dataset was extracted by the MoHW and fully anonymized before sharing with the study investigator, as a result patient consent was waived.

### Sample population

The analytical sample consisted of 8147 PLHIV, 15 years and older, who initiated HIV treatment between January 2015 and December 2019.

### Data analysis and variable definition

Bivariate analysis was used to assess routinely collected categorized demographic and clinical variables related to the pre-defined outcome:

Timing of ART initiation based on the time interval between the first clinic date and the ART initiation date. If these dates were the same, patients were classified as having same-day ART initiation. Timing of ART initiation /days is reported using six categories (preclinic, same day, 1–7, 8–21, 22–30 and 31+).

The categorical variables assessed were: age group at time of treatment initiation, sex, location by regional health authority (RHA) and first viral load (vl) test status (suppressed <1000 copies/mL or non-suppressed ≥1000 copies/mL) as defined by the current national treatment guidelines [9]. HIV stage at diagnosis (early or late) was also assessed: patients starting ART with baseline CD4 cell count ≧350 cells/mm^3^ were characterized as achieving early diagnosis, while a baseline CD4 <350 cells/mm^3^ was defined as late diagnosis.

All categorical variables with a p-value <0.05 from the bivariate analyses were used in a multivariable logistic regression model to assess the factors associated with the binary primary outcome; ART initiation (31+ days vs. same day initiation). We report adjusted odds ratios and 95% confidence intervals. Data was analysed using *R Programme*, version 3.5.3 [21].

## Results

### Data summary

Persons initiating treatment were more likely to be female (n = 4408, 54%), 20–39 years (n = 4407, 54%) and located in the SouthEast Region (SERHA (n = 4140, 51%) (Table 1). Overall, there was a 39% increase in PLHIV starting HIV treatment between 2015–2019, but there was minimal or no increase between 2017–2019. Most persons on treatment were diagnosed at an early HIV stage (n = 4430, 60%). ART initiation occurred mostly at 31+ days after the first clinic visit (n = 3666, 45%) or on the same day (3461, 43%). The median time to ART initiation was 15 days. 53% of persons were not virally suppressed at the first vl test (n = 3713).

**Table 1:** Demographic and clinical characteristics for the analytical sample of all PLHIV on HIV treatment, from 2015–2019 in Jamaica (N = 8147).

Variables			
	Levels	N	%
**Sex**			
	Female	4408	54
	Male	3739	46
**Age group at ART initiation/years**			
	Mean(sd)	38(13)	
	15–19	400	5
	20–39	4407	54
	40+	3340	41
**Regional Health Authority (RHA)**			
	South East (SERHA)	4140	51
	Western (WRHA)	1875	23
	North East (NERHA)	1123	14
	Southern (SRHA)	1009	12
**ARV initiation year**			
2015		1106 14	
2016		1500 18	
2017		1838 23	
2018		1821 22	
2019		1882 23	
**HIV stage at diagnosis**			
	early diagnosis (CD4 >350 cells/mm3)	4430	60
	late diagnosis (CD4 ≤350 cells/mm3)	2941	40
Timing of ART initiation /days^b^			
	Median time of ART initiation(IQR)	15 (449.5)	
	preclinic	277	3
	same day	3461	43
	1–7	201	3
	8–21	282	3
	22–30	260	3
	31+	3666	45
Viral load status at first viral load test^e^			
	Median first viral load result (IQR)^f^	1655(35663)copies/mL	
	non-suppressed	3713	53
	suppressed	3312	47

a) Percentages have been rounded up.

b) Timing of ART initiation/days is the time from first clinic date to ART initiation.

c) Based on the first viral load test result, followed by categorization (suppressed <1000 copies/mL or non-suppressed ≥1000 copies/mL).

d) IQR is reported as the difference between Q3 and Q1.

### Timing of ART initiation

Only 3% (n = 277) of the sample had preclinic ART initiation (Table 2). For post clinic ART initiators, most females (49%, n = 2161) initiated treatment 31+ days after the first clinic visit), while 46% of males (n = 1728) started on the same day. Persons who initiated ART in 2015–2017, more frequently started ART later at 31+days while persons who started treatment in 2018–2019 were more frequently same day ART initiators.

**Table 2:** Demographic and clinical characteristics of PLHIV by timing of ART initiation, for 2015–2019 (N = 8147).

		Timing of ARV initiation/days^b^ ^n(%)^						
		Pre Clinic (n = 277)	Post Clinic (n = 7870)					
Variables	Levels		same day	1–7	8–21	22–30	31+	p
Sex	Female	156(4%)	1733 (39%)	91(2%)	139 (3%)	128 (3%)	2161 (49%)	0.001
	Male	121(3%)	1728 (46%)	99(3%)	143 (4%)	132 (4%)	1505 (40%)	
Age group	15–19	21(5%)	226 (56%)	15(4%)	15(4%)	17(4%)	106 (27%)	<0.001
	20–39	131(3%)	1817 (41%)	111(3%)	151(3%)	123(3%)	2074 (47%)	
	40+	125(4%)	1418 (42%)	75(2%)	116(4%)	120(4%)	1486 (44%)	
RHA	NERHA	28(3%)	445 (40%)	58 (5%)	72(6%)	54 (5%)	466 (41%)	<0.001
	SERHA	133(3%)	1734 (42%)	88(2%)	128(3%)	136 (3%)	1921 (47%)	
	SRHA	22(2%)	436 (43%)	22(2%)	23(2%)	17(2%)	489 (49%)	
	WRHA	94(5%)	846 (45%)	33(2%)	59(3%)	53(3%)	790 (42%)	
ARV year	2015	74(7%)	409 (37%)	27(2%)	33(3%)	29(3%)	534 (48%)	<0.001
	2016	55(4%)	495 (33%)	29(2%)	48(3%)	50(3%)	823 (55%)	
	2017	67(4%)	700 (38%)	37(2%)	61(3%)	49(3%)	924 (50%)	
	2018	41(2%)	893 (49%)	38(2%)	57(3%)	65(4%)	727 (40%)	
	2019	40(2%)	964 (51%)	70(4%)	83(4%)	67(4%)	658 (35%)	
CD4 status^c^	EARLY	101(2%)	1418 (32%)	82(2%)	107(2%)	111(3%)	2611 (59%)	<0.001
	LATE	142(5%)	1498 (51%)	95(3%)	146(5%)	124(4%)	932 (32%)	
Viral load status^d^	Suppressed	129(4%)	1477 (45%)	82(2%)	118(4%)	131(4%)	1375 (41%)	<0.001
	Non-suppressed	107(3%)	1329 (36%)	81(2%)	123(3%)	102(3%)	1965 (53%)	

a) Percentages have been rounded up and reflect row category proportions.

b) Timing of ART initiation/days is the time from first clinic date to ART initiation.

c) Based on baseline CD4 test results: Patients starting ART with CD4 cell count ≧350 cells/mm3 were characterized as achieving early diagnosis, while CD4 <350 cells/mm3 was defined as late diagnosis.

d) Based on the first viral load test after initiating treatment, followed by categorization according to the first viral load test results at that time (suppressed <1000 copies/mL or non-suppressed ≥1000 copies/mL).

The proportion of PLHIV with same day ART initiation increased each year from 37% in 2015 to 51% in 2019 (Table 2) and there was a decrease in ART initiation at 31+ days from 48% to 35% within the same time period. Most persons (n = 2611, 59%) with early HIV diagnosis, initiated ART at 31+ days while 51% (n = 1477) of those with late diagnosis were same day treatment initiators. 45% of persons who were virally suppressed at the first vl test (n = 1477) started ART on the same day, while 53% (n = 1965) of those found to be non-suppressed started at 31+days.

### Factors associated with ART initiation (same day initiation vs 31+ days)

**Fig 1:**
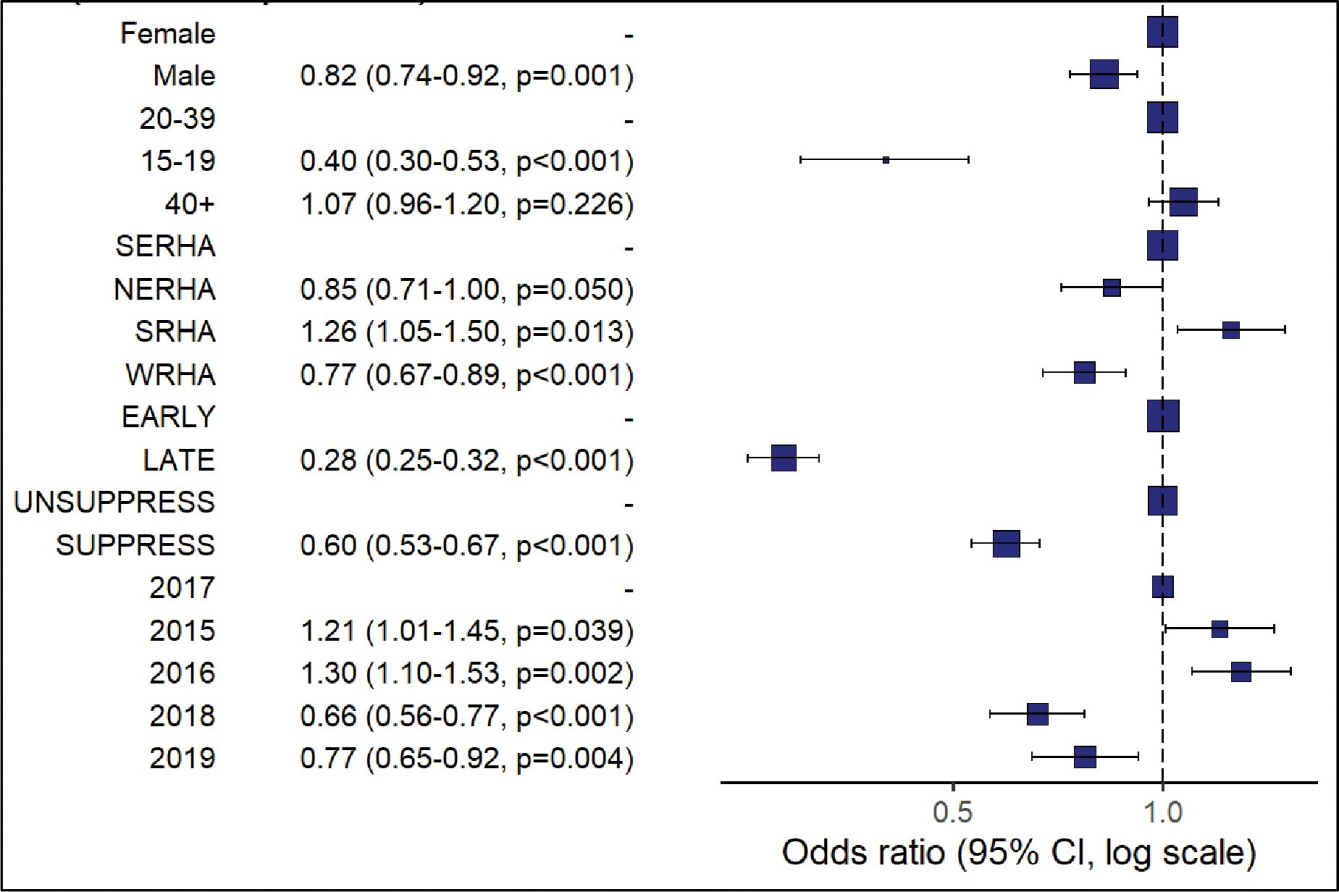
Results of the logistic regression analysis to assess association of PLHIV demographic and clinical variables with ART uptake (same day vs 31+days ART initiation).

Same day ART initiation was associated with males compared to females (aOR = 0.82, CI = 0.74–0.92), 15–19 years old compared to 20–39 years olds (aOR = 0.39, CI = 0.30–0.52), persons located in the WRHA compared to those from SERHA (aOR = 0.79, CI = 0.69–0.90), late HIV diagnosis (aOR = 0.3, CI = 0.27–0.33) and viral suppression at the first viral load test (aOR = 0.6, CI = 0.53–0.67) (Fig 1). Late ART initiation (31+days) was associated with initiation years 2015 (aOR = 1.21, CI = 1.01–1.45) and 2016 (aOR = 1.30, CI = 1.10–1.53), while 2018 (aOR = 0.66, CI = 0.56–0.77) and 2019 (aOR = 0.77, CI = 0.65–0.92) where associated with same day initiation compared to 2017.

## Discussion

Our study population has slightly more females (54%) than males (46%). The majority of PLHIV (60%) are diagnosed early (CD4 ≥ 350); similar proportions initiate treatment on the same day and at 31+ days though same day treatment increased in 2018; and 47% were virally suppressed at their first viral load test.

The overall proportion of PLHIV with same day ART initiation increased from 37% in 2015 to 51% in 2019. However, from 2015–2017 the proportions were similar and the increase occurred in 2018, one year after the “Treat All” strategy was implemented. This suggests that the strategy was successful, though in 2019 over one third (35%) of PLHIV still preferred to wait 31+ days before starting ART. PLHIV with a late diagnosis and therefore possibly symptomatic, were more likely to initiate treatment early. This means the sickest patients were being treated with urgency which did yield results of majority achieving a suppressed viral load at the first viral load test.

Same day ART initiators were more likely to be male, 15–19 years old, located in the WRHA, had a late HIV diagnosis and virally suppressed at the first viral load test. Persons starting ART in 2018 and 2019 were also more likely to start treatment same day. So, a year after implementation of the new strategy, results were becoming evident. Males poor health seeking behaviour may explain late diagnosis which could result in clinical symptoms that prompt immediate treatment initiation. Same day ART initiation is a key goal of the Treat All strategy and while this is important to improving HIV outcomes, ensuring persons are also ready to start treatment is critical [22]. Evidence suggests rapid initiation of ART could lead to increased loss to follow-up because of insufficient time to accept and disclose HIV status and to prepare for lifelong treatment [11]. Persons initiating ART later (31+ days after the first clinic visit) were more likely to start treatment in 2015 and 2016 compared to 2017.

At the regional health level, same day initiation was more likely in the Northeast and Western Region compared to Southeast Region while late initiation was more likely in the Southern Region. This means implementation of the strategy may not have been consistent across the country and possibly delayed in the Southern Region. Increasing access to ART requires expanding several services and ensuring readiness at all levels of care.

There was an overall increase in PLHIV starting treatment from 2015–2019, going from 1106 persons in 2015 and 1500 persons in 2016 to approximately 1800 persons starting treatment each year from 2017–2019. So, in conjunction with the scale up of same day treatment, the number of persons starting treatment also increased but there was no appreciable change for a three-year period. Loss to care between diagnosis and ART retention has been the most common problem in delivery of HIV treatment services [23] and Jamaica is no exception. In 2019, there was an estimated 32,000 PLHIV in Jamaica and 16,640 of those are retained on ART [6]. So, although same day treatment was more likely, retention was an issue and a primary factor in Jamaica not achieving the UNAIDS target in 2020.

We acknowledge there are demand side factors that affect patients’ ability to access treatment, such as social challenges but there is a lack of research exploring supply side factors which can affect service delivery, patient management and bridge the analysis gap. This has become particularly relevant within the context of the COVID-19 pandemic which saw resources being diverted or slowed [24]. It should not be assumed that poor retention only occurs as a result of a patient’s negligence or challenges when there may also be system challenges [25]. Overall, published research related to ART retention, adherence and other treatment outcomes in the local setting are limited.

We recognize the limitations of the data. Improved accuracy is expected if the data was normally distributed [26,27] but medians are reported to account for skewness of the data. The data did not allow us to measure the time retained on ART/adherence or time retained in care. We are unable to relate viral load results to adherence. Table 2. shows 3% of persons received ART preclinic visit, indicating these persons were receiving treatment at clinics not included in the public site data, possibly hospitals. However, the sample size is large and the majority of HIV patients are currently receiving care in the public sector, which limits selection bias. Also, the regression model was strong and produced a c-statistic of 70% accuracy for factors associated with ART initiation.

## Conclusion

Our study shows that same day ART initiation has increased between 2015 and 2019 however it remains too low. Same day initiation was associated with males, late HIV diagnosis, ART initiation in 2018–2019 and virally suppression at the first viral load test while late ART initiation was associated with initiation years 2015–2016. In order to achieve the UNAIDS targets, there is a need to also increase the number of diagnosed PLHIV retained on treatment. Further studies should be conducted to understand important challenges to accessing HIV treatment as well as differentiated care models to improve retention.

## Abbreviations

ART: antiretroviral therapy
PLHIV: persons living with HIV
NERHA: Northeast Regional Health Authority
NGO: non-governmental organization
RHA: Regional Health Authority
SERHA: Southeast Regional Health Authority
SRHA: Southern Regional Health Authority
WRHA: Western Regional Health Authority
